# Delivering Patient Data to Patients Themselves

**DOI:** 10.5334/egems.267

**Published:** 2018-06-26

**Authors:** Jessica S. Ancker

**Affiliations:** 1 Weill Medical College of Cornell University, US

**Keywords:** patient-centered clinical decision support, clinical decision support, PCOR, CDS

## Abstract

Physicians need nearly a decade of training to understand complex patient data such as laboratory tests and genomic data. How can these data possibly be delivered to patients in ways that they can understand and use?

The beginnings of an answer can be found in the first new articles in *Better Decisions Together, eGEMs*’s recently launched special section on patient-centered clinical decision support:

- “The Imperative for Patient-Centered Clinical Decision Support” by Laura Marcial et al.,
- “Generation and Implementation of a Patient-Centered and Patient-Facing Genomic Test Report in the EHR” by Jessica Goehringer et al., and
- “Methods for Patient-Centered Interface Design of Test Result Display in Online Portals” by Daniel Nystrom et al.

The first of these papers (Marcial et al.) helps describe the context for our new *Better Decisions Together* section and introduces the Patient-Centered Clinical Decision Support (PCCDS) Learning Network—an initiative supported by the Agency for Healthcare Research and Quality (AHRQ) to inform and connect PCCDS stakeholders and broadly advance PCCDS initiatives and their value in making care more patient centered.

Meanwhile, the two following papers in this inaugural set both suggest that the way forward is to engage patients integrally in conceptualizing, designing, and testing new information tools. Patient engagement should go beyond just asking patients to comment on designs made by medical professionals or software engineers. Instead, the best results come when patients are invited to co-create tools or reports they themselves would find useful for their own needs.

These articles also suggest some additional important lessons for us in designing patient-centered clinical decision support.

Our first lesson is that we should not underestimate patients’ needs for information and their interest in learning. “Simplifying” materials for patients often means providing more information, not less. Some of this additional information will be background, which is necessary for anybody learning a new concept. Some of it will be context, to help patients see why the data is relevant to decisions they might need to make. So it is not surprising that in our first *Better Decisions Together* original research articles, the patient participants recommended including additional information in the reports, often including material that the medical experts had suggested might constitute “too much information.” Goehringer and colleagues also found that patients did not want to replace technical jargon in genomic reports altogether with more familiar terms. Instead, they wanted to the technical terms included together with their definitions, so they could learn the vocabulary for future reference.

A second lesson is to avoid reinventing the wheel. Both teams of researchers applied lessons from existing literature about patient-facing information to develop the early prototypes. That meant that patients and informatics teams did not need to waste time and resources reestablishing known principles of communication, but could instead focus on specific pragmatic issues about extending what is known and adapting it to new situations.

This excellent work doesn’t mean that medical organizations should withhold raw data, reports, and images from patients who want them. It does, however, begin to show the possibilities when that raw data is transformed to be more patient-centered.

Genuinely patient-centered clinical decision support will depend in part on finding new and better ways to get complex patient-specific data and test results into the hands of patients, in formats they can understand, with additional resources they can use to educate themselves further. These inaugural papers in *Better Decisions Together* highlight this important work and some crucial steps toward that goal.

